# The cytotoxicity effect of 7-hydroxy-3,4-dihydrocadalene from *Heterotheca inuloides* and semisynthetic cadalenes derivates towards breast cancer cells: involvement of oxidative stress-mediated apoptosis

**DOI:** 10.7717/peerj.15586

**Published:** 2023-06-20

**Authors:** Alan Mendoza-Fuentes, Elena González-Burgos, Omar Emiliano Aparicio Trejo, Guillermo Delgado-Lamas, José Luis Rodríguez-Chávez, José Pedraza-Chaverri, M. Pilar Gómez-Serranillos, Daniela Araiza-Olivera

**Affiliations:** 1 Institute of Chemistry, Universidad Nacional Autónoma de México, México City, México; 2 Department of Pharmacology, Pharmacognosy and Botany, Faculty of Pharmacy, University Complutense of Madrid, Madrid, Spain; 3 Departament of Biology, Faculty of Chemistry, Universidad Nacional Autónoma de México, México City, México; 4 Fox Chase Cancer Center, Philadelphia, United States

**Keywords:** Cytotoxicity, Cadalenes, Breast cancer, Oxidative stress, Apoptosis

## Abstract

**Background:**

*Heterotheca inuloides*, traditionally employed in Mexico, has demonstrated anticancer activities. Although it has been proven that the cytotoxic effect is attributed to cadinane-type sesquiterpenes such as 7-hydroxy-3,4-dihydrocadalene, the mechanism of action by which these agents act in tumor lines and their regulation remain unknown. This study was undertaken to investigate for first time the cytotoxic activity and mechanism of action of 7-hydroxy-3,4-dihydrocadalene and two semi-synthetic cadinanes derivatives towards breast cancer cells.

**Methods:**

Cell viability and proliferation were assayed by thiazolyl blue tetrazolium bromide (MTT) assay and Trypan blue dye exclusion assay. Cell migration measure was tested by wound-healing assay. Moreover, the reactive oxygen species (ROS) and lipid peroxidation generation were measured by 2′,7′-dichlorofluorescein diacetate (DCFH-DA) assay and thiobarbituric acid reactive substance (TBARS) assay, respectively. Furthermore, expression of caspase-3, Bcl-2 and GAPDH were analyzed by western blot.

**Results:**

The results showed that 7-hydroxy-3,4-dihydrocadalene inhibited MCF7 cell viability in a concentration and time dependent manner. The cytotoxic potency of semisynthetic derivatives 7-(phenylcarbamate)-3,4-dihydrocadalene and 7-(phenylcarbamate)-cadalene was remarkably lower. Moreover, *in silico* studies showed that 7-hydroxy-3,4-dihydrocadalene, and not so the semi-synthetic derivatives, has optimal physical-chemical properties to lead a promising cytotoxic agent. Further examination on the action mechanism of 7-hydroxy-3,4-dihydrocadalene suggested that this natural product exerted cytotoxicity *via* oxidative stress as evidenced in a significantly increase of intracellular ROS levels and in an induction of lipid peroxidation. Furthermore, the compound increased caspase-3 and caspase-9 activities and slightly inhibited Bcl-2 levels. Interestingly, it also reduced mitochondrial ATP synthesis and induced mitochondrial uncoupling.

**Conclusion:**

Taken together, 7-hydroxy-3,4-dihydrocadalene is a promising cytotoxic compound against breast cancer *via* oxidative stress-induction.

## Introduction

Reactive oxygen species (ROS) are a heterogeneous group of highly reactive ions and molecules derived from molecular oxygen that include species with unpaired (or radical species) and paired (or species non-radicals) electrons (Liou & Storz, 2010). In cells, ROS are products of synthetic and degradation pathways. Mitochondrial electron transport chain is the largest ROS intracellular source (Saybaşili et al., 2001). Apart from electron transport chain, other endogenous sources have been identified such as xanthine oxidases, NADPH oxidases, lipoxygenases, and cytochrome P450 (Andreyev, Kushnareva & Starkov, 2005; Andreyev et al., 2015). Additionally, ROS generation can be facilitated by exogenous factors such as radiation, chemical agents, heavy metals, and contaminants (Bhattacharyya et al., 2014).

The balance between ROS production and elimination is critically important; it has been related to numerous biological processes and diseases (Alfadda & Sallam, 2012; Görlach et al., 2015; Manoharan et al., 2016). ROS act as secondary mediators in intracellular signaling to regulate physiological and biological responses (Sena & Chandel, 2012; Schieber & Chandel, 2014). However, oxidative stress condition leads to ROS accumulation that react with proteins, lipids (mainly cell membranes) and DNA, and interrupt cellular processes (Schumacker, 2015).

Breast cancer is the most common type of cancer and first cause of cancer death in women (Ferlay et al., 2020). Cancer is one of the diseases caused by ROS unbalance. Malignant cells have higher ROS levels compared to normal counterparts; this is related to faulty mitochondrial oxidative metabolism (Tafani et al., 2016). Tumorigenic events can increase intracellular ROS levels, promoting tumor progression. However, excessive increases of ROS levels can induce cell cycle arrest, senescence, or trigger apoptosis in cancer cells that lead to cell death (Glasauer & Chandel, 2014; Galadari et al., 2017). Therapeutic strategies that increase ROS generation and/or decrease antioxidant defense can selectively kill tumor cells by suppressing tumor growth and progression. The cytotoxic specificity with which ROS act on malignant cells lies in their redox balance since non-tumor cells have a different redox environment and are less sensitive to redox manipulation. Therefore, promoting ROS levels production in cancer cells to induce apoptosis and unbalance the homeostasis of ROS/antioxidants is a very promising therapeutically strategy to prevent and treat cancer (Matés & Sánchez-Jiménez, 2000). In this sense, there are many unexplored natural products with pro-oxidants properties which are a promising source of novel anticancer bioactive compounds of growing interest (Cragg & Newman, 2013; Greenwell & Rahman, 2015; Jain et al., 2016).

*Heterotheca inuloides* Cass (Asteraceae family), known commonly as Mexican arnica, has been widely employed in traditional medicine in Mexico. It is one of the plants included in the Herbolary Pharmacopeia (Secretariat of Health of Mexico, 2008). Dried inflorescences are mainly used to treat inflammatory-related diseases such as rheumatism, contusions, and skin problems (Rodríguez-Chávez et al., 2017). Moreover, *H. inuloides* and its secondary metabolites have shown antimicrobial, antinociceptive, antioxidant and anticancer activities (Coballase-Urrutia et al., 2010; Rocha-González et al., 2010; Rodríguez-Chávez et al., 2015a, 2015b; Egas et al., 2018). From multiple constituents found in *H. inuloides* such as polyacetylenes, sterols and flavonoids, most of the ethnomedicinal properties are attributed to sesquiterpenes which are characterized by having a cadinane skeleton. From all these derivatives, the cadalenes 7-hydroxy-3,4-dihydrocadalene and 7-hydroxycadalene presented cytotoxic effect against tumoral cells (Kubo et al., 1996). Cytotoxicity studies of these compounds are still very limited and focused basically on assessing their effect on cell viability. The mechanism of toxicity has not yet been studied (Rodríguez-Chávez et al., 2015b; Egas et al., 2018, 2017).

**Figure 1:**
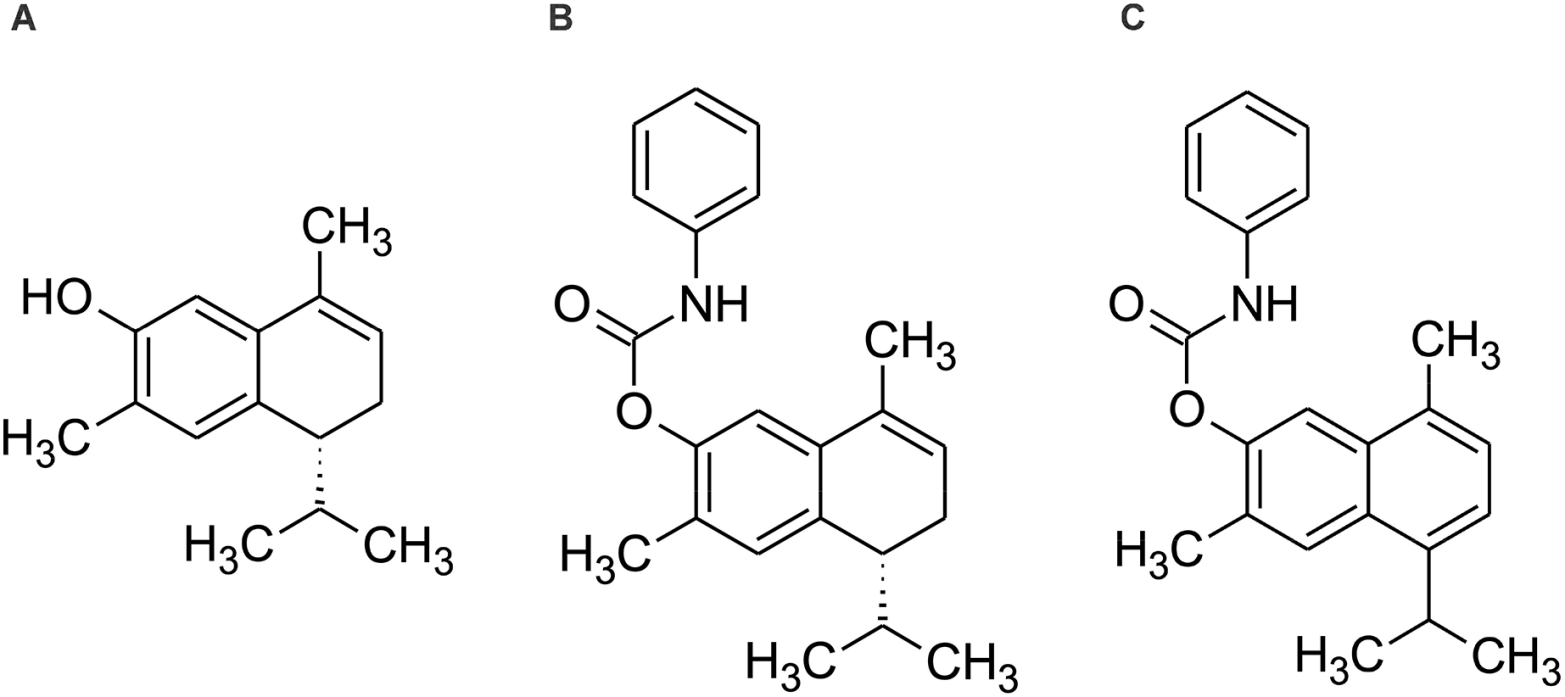
Chemical structure of (A) 7-hydroxy-3,4-dihydrocadalene, (B) 7-(phenylcarbamate)-3,4-dihydrocadalene and (C) 7-(phenylcarbamate)-cadalene.

Therefore, the aim of the present study is to evaluate the cytotoxic effect and elucidate the mechanism of action of 7-hydroxy-3,4-dihydrocadalene from *Heterotheca inuloides* and the semi-synthetic cadinanes derivatives 7-(phenylcarbamate)-3,4-dihydrocadalene and 7-(phenylcarbamate)-cadalene against MCF7 breast cancer cell line (Fig. 1).

## Materials and Methods

### Reagents and chemicals

RPMI 1640 medium, gentamicin, trypsin, phosphate-buffered saline (PBS) and fetal bovine serum (FBS) were acquired from Gibco (Invitrogen, Carlsbad, CA, USA). Isolated compounds 7-hydroxy-3,4-dihydrocadalene (>98.0%), 7-(phenylcarbamate)-3,4-dihydrocadalene (77% yield) and 7-(phenylcarbamate)-cadalene (79% yield) were generously provided by Dr. Guillermo Delgado Lamas (Instituto de Química, UNAM). Their isolation or preparation and structural characterization are described by Egas et al. (2017).

Dimethyl Sulphoxide (DMSO), H_2_O_2_, 2′,7′-Dichlorofluorescein Diacetate (DCFH-DA), 3-(4,5-Dimethyl-2-thiazolyl)-2,5-diphenyl-2H-tetrazolium bromide (MTT), Bovine Serum Albumin (BSA), 2-thiobarbituric acid, antimycin A, oligomycin, rotenone, RIPA buffer, phosphatase inhibitor cocktails, bicinchoninic acid reagent, Tris Buffered Saline-Tween 20, GAPDH antibody (G-8795), caspase 3 (sc-7148) and Bcl-2 (sc-492) antibodies were purchased from Sigma-Aldrich (St. Louis, MO, USA). Protease inhibitor cocktail from Roche (Lewes, UK). Polyvinylidene difluoride (PVDF) membranes from GE Healthcare (Buckinghamshire, UK).

### Cell culture

MCF7 (#HTB-22) cell line, derived from human breast adenocarcinoma, was obtained from the American Type Culture Collection (ATCC®, Manassas, VA, USA). It was maintained under standard conditions: RPMI-1640, 2.0 g/L NaHCO_3_, supplemented with 10% FBS and gentamicin 50 mg/mL.

HEK 293 (CRL-1573) cell line, human embryonic kidney, was obtained from ATCC® (Manassas, VA, USA). It was grown in DMEM medium (Caisson Laboratories, Inc., Smithfield, UT, USA) with 10% v/v phenol red of inactivated FBS, 44 mM NaHCO_3_, 1 mM sodium pyruvate and penicillin 100 I.U.−100 μg/mL streptomycin−25 ng/mL amphotericin B, pH 7.4. Both cell lines were incubated at 37 °C and 5% CO_2_.

### MTT assay: cell viability (mitochondrial activity) determination

Cells were treated with isolated compounds (0.1, 1, 5, 10, 25, 50 and 100 µM) for 24, 48 and 72 h. After treatments, culture media was replaced by MTT (100 μL) dissolved in RPMI media (0.33 mg/mL final concentration) per well and incubated at 37 °C with 5% CO_2_ for 1 h in dark. After incubating, supernatant was removed and replaced with DMSO to dissolve formazan crystals. Absorbance value was measured in a microplate reader (SPECTROstar Nano; BMG Labtech, Ortenberg, Germany) at 570 nm. IC_25_ and IC_50_ values were determined using GraphPad Prism 7.00 (GraphPad Software, La Jolla, CA, USA). Percentage of cell viability was calculated in comparison with control cells (Mosmann, 1983).

### *In silico* assay

DataWarrior software (v4.7.2; https://openmolecules.org/datawarrior/) was used to predict the physicochemical properties molecular weight (M_W_), partition coefficient molecular weight (logP), aqueous solubility distribution coefficient at pH = 7.4 (logD), number of hydrogen donors (HBD), number of hydrogen bond acceptors (HBA), topological polar surface area (TPSA) and number of links that can be rotated (n_Rot_) of isolated compounds.

### Trypan blue dye exclusion assay: cell proliferation quantification

Proliferation was evaluated using trypan blue dye exclusion assay at different incubation times (0, 2, 4, 8, 24, 48 and 72 h) employing IC_50_ (55.24 μM) and IC_50_/2 (27.62 μM) value of 7-hydroxy-3,4-dihydrocadalene. Cells were trypsinized, centrifuged and stained with trypan blue dye (0.4% w/v in PBS). The number of viable cells and dead cells were counted on a Neubauer hemocytometer under an inverted microscope. The percentage of cell proliferation was calculated by comparing with control cells.

### Wound-healing assay: cell migration measure

Cells were seeded in triplicate in six-well plates under standard conditions until 90–100% confluence was reached, followed by wounding the monolayer by scratching with a micropipette tip, then cells were treated with H_2_O_2_ 1.0 mM, IC_50_ (55.24 μM) and IC_50_/2 (27.62 μM) of 7-hydroxy-3,4-dihydrocalene compound in RPMI medium supplemented with 1% FBS. Wound areas were monitored at four different times (0, 24, 48, and 72 h) using the Cytation 5 cell imager (BioTek Instruments, Inc., Winooski, VT, USA). To determine the migration rate of the cells, the wound areas were quantified using the ImageJ software, (Schneider, Rasband & Eliceiri, 2012). The variation between the percentage of cell migration (as a function of time) was calculated using the following formula:

${\rm \% \;Migration = 100 - [(Area\; of\; injury\; time\; t / Area\; of\; injury\; time \;zero)\;(100)]}$

### 2′,7′-dichlorofluorescein diacetate (DCFH-DA) assay: intracellular ROS detection

Intracellular ROS were detected by fluorescent compound DCFH-DA. Cells were treated with 1.0 mM H_2_O_2_, IC_50_ (55.2 μM) and IC_50_/2 (27.6 μM) of 7-hydroxy-3,4-dihydrocadalene for 48 h. Then, culture media was replaced with DCFH-DA (20 μM dissolved in glucose 1.8 mg/mL PBS) per well and incubated in the dark for 30 min. DCF fluorescence intensity was measured using an FLx800 microplate fluorescence reader (Bio-Tek Instruments, Inc., Winooski, VT, USA) at 480 and 530 nm excitation and emission wavelengths, respectively (LeBel, Ischiropoulos & Bondy, 1992).

### Pellet harvest and total extract

Mechanical scraping was employed to collect adherent MCF7 cells after treatments. Then, cells were centrifugated (1,500 rpm for 5 min at 37 °C) and supernatants were removed. Pellets were lyzed adding lysis buffer (25 mM TrisHCl, pH 7.4, 150 mM NaCl, 1 mM EDTA, 0.1% Triton X-100, 1 mg/mL leupeptin, 0.5 mM PMSF and 1 mg/mL pepstatin) and put on ice for 20 min. Finally, cell lysates were centrifugated (2,500 rpm for 10 min at 4 °C) and supernatants were collected to following assays.

### Bicinchoninic acid assay: protein measure

Total protein concentrations were measured as described by Smith et al. (1985). BCA/CuSO_4_ reactive solution (1:20) was added to each cell lysates and incubated in the dark for 30 min at 37 °C. Absorbance was measured with a SPECTROstar Nano microplate reader (BMG Labtech, Ortenberg, Germany) at a wavelength of 550 nm.

### TBARS assay: lipid peroxidation determination

Lipid peroxidation was measured colorimetrically as described by Uchiyama & Mihara (1978). Cell pellets, stored at −80 °C, were thawed at room temperature and then, they were homogenized in thiobarbituric acid reactive substances (TBARS) solution (28.2 mM 2-TBA in 18.5 mL of TCA 16%-HCl 0.27 N). Then, the mixtures were heated for 10 min at 100 °C, and subsequently cooled in ice to stop the reaction. Supernatants of each sample were separated by centrifugation (3,000 rpm for 10 min at 4 °C) and immediately loaded into a SPECTROstar Nano microplate reader (BMG Labtech, Ortenberg, Germany) for absorbance measurement at 530 nm. TBARS values were expressed as nmol/mg protein.

### Caspase-3/9 activity assay

Fluorometric assays of caspase-3 and caspase-9 enzymes were performed in accordance with the instructions provided by ALEXIS Biochemicals kits (Enzo Life Sciences Inc., San Diego, CA, USA). This enzymatic assay was based on hydrolysis of the peptide substrates of caspases-3/9 (Ac-DEVD 20 μM and Ac-LEHD 20 μM, respectively) conjugated to a fluorochrome (7-amido-4-methylcoumarin or AMC) at the terminal carbon of Asp, resulting in the release of the fluorescent motif. Cell lysates (20 μg) were used for each treatment. After incubation for 1 h at 37 °C, fluorescence was determined using a FLx800 microplate reader at excitation and emission wavelengths of 380 and 460 nm, respectively.

### Immunoblotting

Western blot assays were performed using standard techniques. Primary antibodies were anti-caspase-3, Bcl-2 and GAPDH from Santa Cruz Biotechnology (Santa Cruz Biotechnology, Inc., Dallas, TX, USA). The secondary antibody was anti-rabbit IgG, HRP-linked (Antibody #7074; Cell Signaling Technology, Danvers, MA, USA).

### Cell respirometry

The oxygen consumption experiments were performed using a high resolution respirometry equipment O2k meter (Oroboros Instruments, Innsbruck, Austria) described by Aparicio-Trejo et al. (2019). The respiratory parameters were defined as: (A) Routine respiration, corresponding to oxygen consumption in presence only of cells in culture medium. (B) Leak of the respiration, corresponding to cellular oxygen consumption in presence of 15 μM oligomycin. (C) RCI corresponding to the ratio basal/leak. (D) Respiration attributable to OXPHOS (P) corresponding to: Routine-Leak. All parameters were corrected by subtracting non-mitochondrial respiration resulting from the addition of 15 μM rotenone and 15 μM antimycin A and normalized by cells number.

### Statistical analysis

All the data were analyzed and plotted using GraphPad Prism software version 7.00 for Windows (GraphPad Software, San Diego, CA, USA) and Microsoft Office Excel version 16.17 for macOS, while the statistical analysis was performed through the one- or two-way ANOVA test of the Tukey, Dunnett or Sidak multiple comparisons tests. The values *p* < 0.05 were considered statistically significant.

## Results

### Effect on cell viability

**Figure 2:**
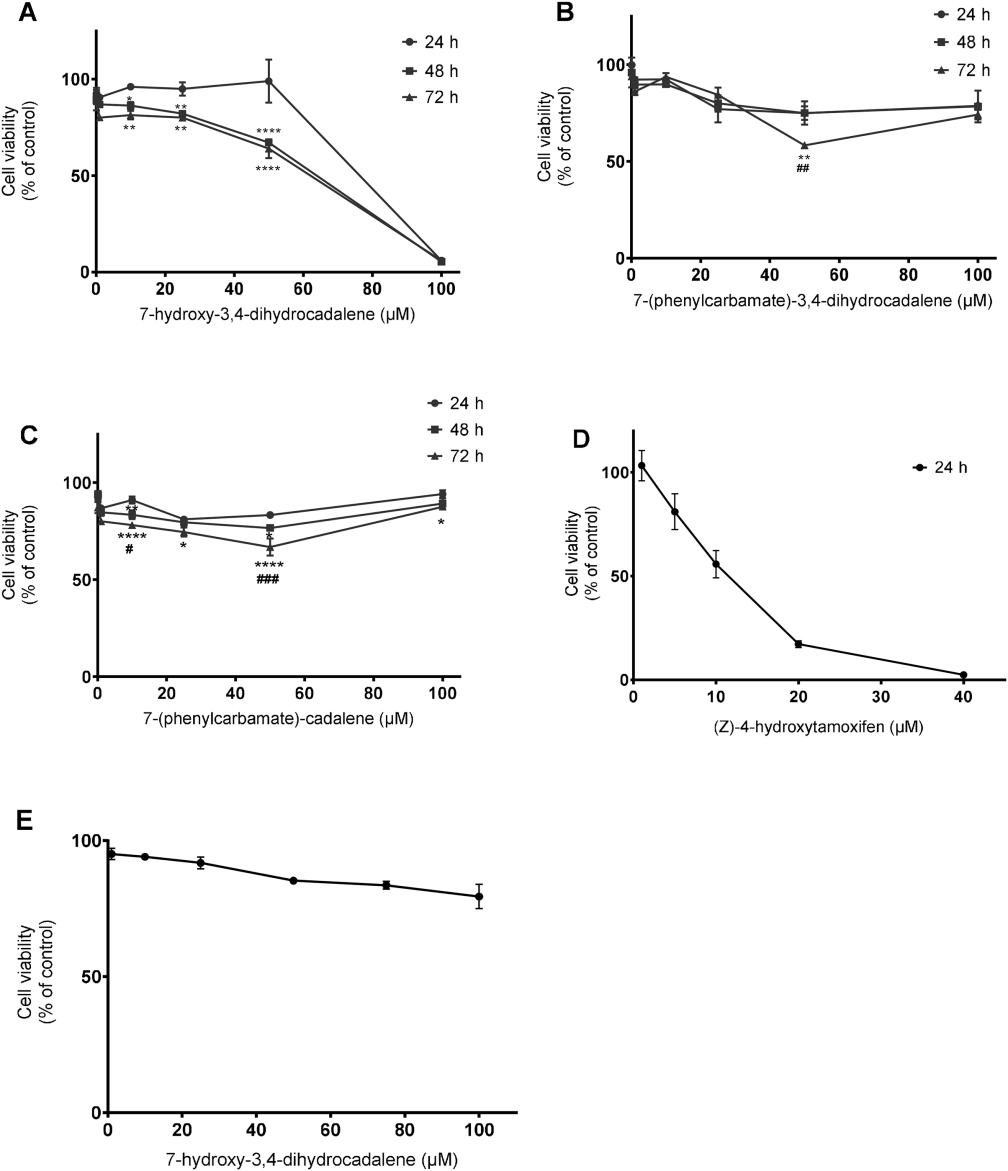
Effect on cell viability. MCF7 human breast cancer cells were treated with different concentrations from 0.1 to 100 µM for 24, 48, and 72 h with: (A) 7-hydroxy-3,4-dihydrocadalene, (B) 7-(phenylcarbamate)-3,4-dihydrocadalene, (C) 7-(phenylcarbamate)-cadalene Moreover, (D) (Z)-4-hydroxytamoxifen at 5, 10, 20, and 40 µM was added to MCF7 for 24 h and (E) human embryonic cells from kidney (HEK 293, control cells) were treated with 7-hydroxy-3,4-dihydrocadalene for 72 h. MTT assay was performed. Results are shown as means ± SD of at least three independent experiments (*P < 0.05, **P < 0.01 and ****P < 0.0001 *vs*. 24 h; ^#^*P* < 0.05, ^##^*P* < 0.01 and ^###^*P* < 0.001 *vs*. 48 h).

Initially, we investigated the effect of 7-hydroxy-3,4-dihydrocadalene from *Heterotheca inuloides* and the semi-synthetic cadinanes derivatives 7-(phenylcarbamate)-3,4-dihydrocadalene and 7-(phenylcarbamate)-cadalene on MCF7 breast cancer cell line. As shown in Fig. 2A, the natural product 7-hydroxy-3,4-dihydrocadalene reduced MCF7 cell viability in a concentration and time-dependent manner. The highest cytotoxicity was found at 48 and 72 h of exposure. Significant differences were observed between treatments at 24 and 48 h and, at 24 and 72 h, but not significant differences between treatments at 48 and 72 h. The inhibitory concentration (IC_50_) values for 7-hydroxy-3,4-dihydrocadalene were 55.24 µM at 48 h and 52.83 µM at 72 h (Fig. 2A). On the other hand, the cytotoxic potency of the semisynthetic derivatives 7-(phenylcarbamate)-3,4-dihydrocadalene and 7-(phenylcarbamate)-cadalene were remarkably much lower than the natural product. As shown in Fig. 2B, the compound 7-(phenylcarbamate)-3,4-dihydrocadalene did not show a clear trend of MCF7 cell viability reduction dependently on the concentration or the time. However, the compound 7-(phenylcarbamate)-cadalene slightly inhibited MCF7 cell growth with different concentrations, 10 and 50 µM at 72 h of exposure (Fig. 2C). It was not possible for the semisynthetic compounds to determine the IC_50_ values under the experimental conditions established in this work.

As a positive control we used (Z)-4-hydroxytamoxifen an estrogen receptor inhibitor (highly expressed in MCF7), obtaining an IC_50_ of 16.50 µM at 24 h (Fig. 2D). Finally, non-tumoral cells from kidney (HEK 293) were treated with 7-hydroxy-3,4-dihydrocadalene for 72 h without effect (Fig. 2E).

### Physicochemical properties with cytotoxic potential

As shown in Table 1, the molecular weight of the three studied compounds was less than 350 Da, being especially lower for the natural product (216.3 g/mol). Moreover, none of the studied compounds have ionizable groups as evidenced the identical values for logP and logD. Furthermore, the values of logS were −4.23 for 7-hydroxy-3,4-dihydrocadalene, −6.65 for 7-(phenylcarbamate)-3,4-dihydrocadalene and −7.47 for 7-(phenylcarbamate)-cadalene. Regarding the number of hydrogen bond donors, this is identical for the three compounds (1), whereas the number of hydrogen acceptors is three for semisynthetic compounds and one for the natural product. There are also differences in TPSA and nRot between the natural product and the semisynthetic compounds.

**Table 1:** Estimation of molecular formula, molecular weight, logP, logS, logD, number of hydrogen bond donors (HBD) and number of hydrogen bond acceptors (HBA), topological polar surface area (TPSA) and number of rotatable bonds have been estimated by using Osiris Data Warrior software (http://www.openmolecules.org/datawarrior/).

	7-hydroxy-3,4-dihydrocadalene	7-(phenylcarbamate)-3,4-dihydrocadalene	7-(phenylcarbamate)-cadalene
Molecular formula	C_15_H_20_O	C_22_H_25_NO_2_	C_22_H_23_NO_2_
Mw (g/mol)	216.323	335.446	333.430
LogP	4.548	6.122	6.699
LogS	−4.227	−6.657	−7.466
LogD	4.55	6.12	6.70
HBA	1	3	3
HBD	1	1	1
TPSA (Α°)	20.23	38.33	38.33
nRot	1	4	4

### Effects on morphological changes and cell migration

**Figure 3:**
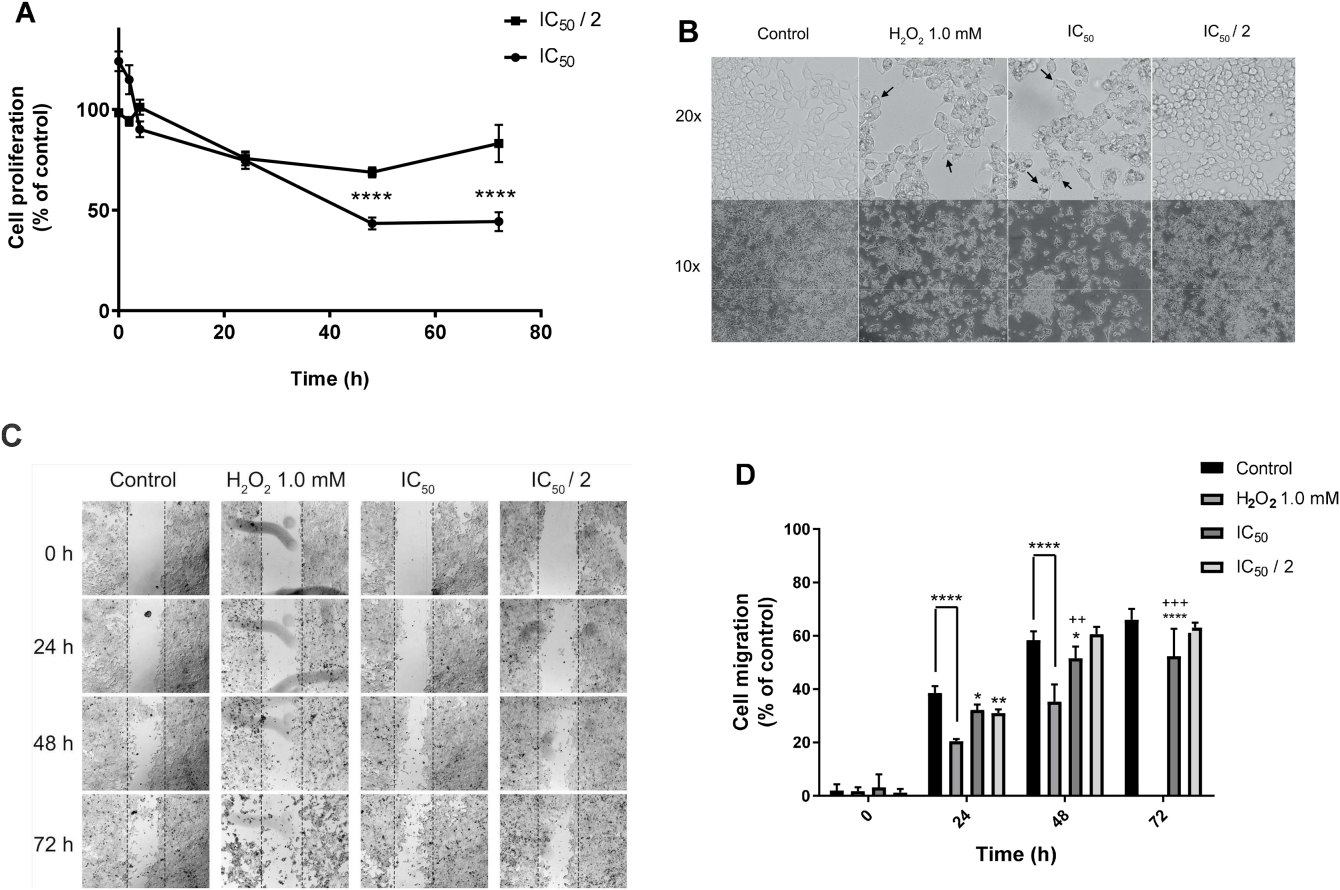
Effect on cell proliferation, morphology, and migration. MCF7 human breast cancer cells were treated with IC_50_ and IC_50_/2 values of 7-hydroxy-3,4-dihydrocadalene for 48 h, and then: (A) Trypan blue assay was performed to measure cell proliferation and (B) cell imaging multi-mode reader Cytation 5 was used to study cell morphology. (C) Wound healing in MCF7 monolayer at 0, 24, 48, and 72 h after treatment (10×). The dotted lines indicate the edge of the initial wound. (D) Cell migration separation area was quantified at different times with each treatment and compared with the percentage of reduction of the initial area (0 h). The results were expressed as a percentage of migration capacity of the cells with respect to time zero of each group. The results are shown as means ± SD of at least three independent experiments (*P < 0.05, **P < 0.01, ****P < 0.0001 *vs*. Control and ^++^*P* < 0.01, ^+++^*P* < 0.001 *vs.* IC_50_/2).

Continuing with the study of the cytotoxic activity of the natural product 7-hydroxy-3,4-dihydrocadalene, we evaluated its effect on MCF7 proliferation and cell morphology with IC_50_ and IC_50_/2 concentrations for 48 h. As shown in Fig. 3A, IC_50_ value significantly reduced cell proliferation in a concentration and time dependent manner. Moreover, morphological changes (cells are rounded off, adhesion capacity decreases, and membrane is blebbing) were observed after treatments with IC_50_ value of 7-hydroxy-3,4-dihydrocadalene and H_2_O_2_ (1 mM as positive control) (Fig. 3B).

The effect of the natural product on cell migration (wound-healing assay) was analyzed using a multimodal plate reader to monitor the motility of the treated cells with 1 mM H_2_O_2_, IC_50_ (48 h) and IC_50_/2 of the natural compound. Fig. 3C illustrates a representative time course of the promotion or inhibition of MCF7 monolayer healing exposed to each treatment, while Fig. 3D shows the quantification of cell migration as a function of time and treatment where IC_50_ of 7-hydroxy-3,4-dihydrocadalene significantly inhibits MCF7 migration at 24, 48 and 72 h of treatment exposition. Apparently after 72 h of treatment with 1 mM H_2_O_2_ cells were completely detached and it was not able to be quantified.

### Effect on reactive oxygen species (ROS) and lipid peroxidation levels

**Figure 4:**
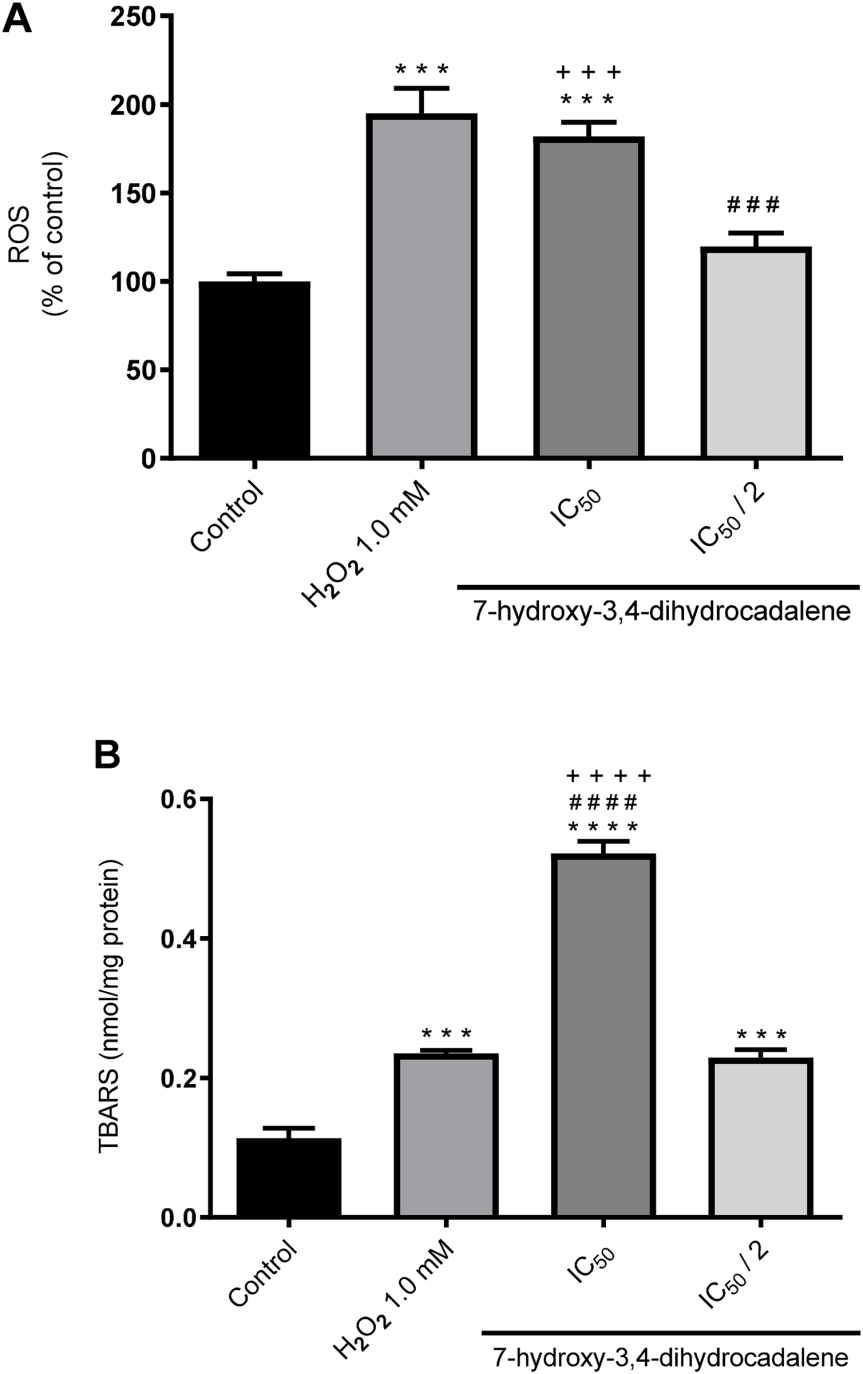
Effect of 7-hydroxy-3,4-dihydrocadalene on: (A) intracellular ROS production and (B) lipid peroxidation. MCF7 cells were treated with IC_50_ and IC_50_/2 values and hydrogen peroxide 1.0 mM as positive control for 48 h, and then lipid peroxidation was measured by TBARS assay. Results are shown as means ± SD of at least three independent experiments (***P < 0.001 and ****P < 0.0001 *vs*. control; ^###^ *P* < 0.001 and ^####^ *P* < 0.0001 *vs*. hydrogen peroxide and ^+++^*P* < 0.001).

Next, we evaluated whether the natural product 7-hydroxy-3,4-dihydrocadalene could generate intracellular ROS using the DCFH-DA assay. H_2_O_2_ was used as positive control (1 mM for 24 h). As shown in Fig. 4A, 7-hydroxy-3,4-dihydrocadalene significantly increased the intracellular ROS levels at IC_50_ concentration (182% compared to control cells) like that of H_2_O_2_ (194.9% compared to control cells). Significant differences were also found between IC_50_ and IC_50_/2 treatments.

Moreover, we explore the effect of 7-hydroxy-3,4-dihydrocadalene on lipid peroxidation using the thiobarbituric acid reactive substance (TBARS) method. As shown in Fig. 4B, lipid peroxidation levels significantly increased at IC_50_ concentration of 7-hydroxy-3,4-dihydrocadalene (0.52 nmol/mg protein) compared to control cells (0.11 nmol/mg protein), IC_50_/2 (0.23 nmol/mg protein) and even the positive control hydrogen peroxide (0.24 nmol/mg protein) (Fig. 4B).

### Effect on cell death through apoptosis

**Figure 5:**
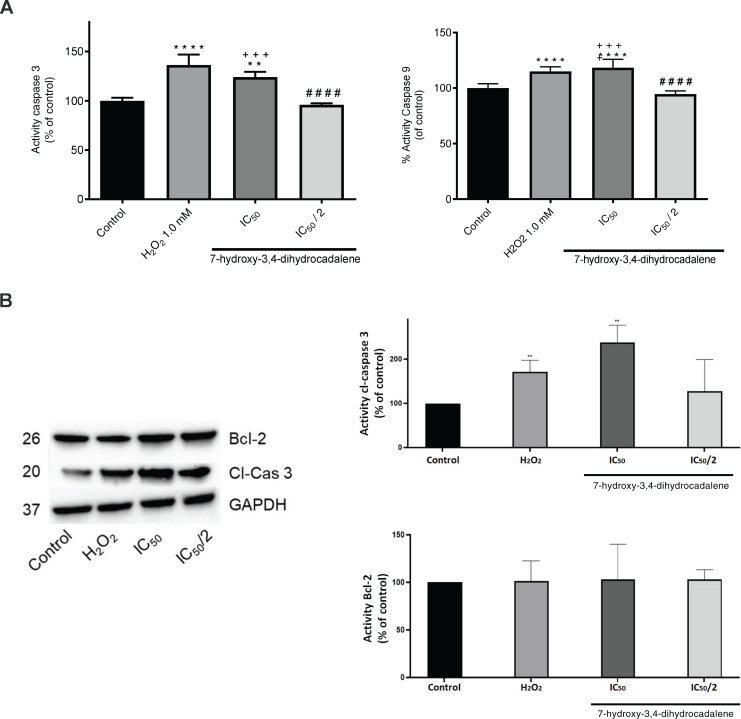
Apoptotic effect of 7-hydroxy-3,4-dihydrocadalene. MCF7 cells were treated with IC_50_ and IC_50_/2 and H_2_O_2_ 1.0 mM as positive control for 48 h. (A) Caspase 3 and caspase 9 activities were measured using the fluorimetric substrate Ac-DEVD-AMC. (B) The cell lysates were analyzed by Western blot for the indicated cleaved-caspase 3, reprobed for Bcl-2 and GAPDH was used as a loading control. Results are shown as means ± SD of three independent experiments (**P < 0.01 and ****P < 0.0001 *vs*. control; ^####^*P* < 0.0001 *vs*. hydrogen peroxide and ^+++^*P* < 0.001 *vs*. IC_50_/2).

To corroborate if the cytotoxic effect of 7-hydroxy-3,4-dihydrocadalene was apoptosis pathway mediated, caspase 3 and caspase 9 were evaluated as the effector enzymes that initiate the point of no return in the apoptotic death cascade. As shown in Fig. 5A, 7-hydroxy-3,4-dihydrocadalene increased significantly both caspase 9 and caspase 3 activities (values of 118.42% and 24.17%, respectively compared to control cells) like the H_2_O_2_ (values of 115.08% for caspase 9 and 136.25% for caspase 3). However, no effect was observed on caspases 3 and 9 at the IC_50_/2 concentration. Moreover, we measured caspase 3 and Bcl-2 levels by immunoblotting after 7-hydroxy-3,4-dihydrocadalene treatments (IC_50_ and IC_50_/2) and H_2_O_2_. The WB analysis in Fig. 5B showed that the expression of caspase 3 with H_2_O_2_ increased and it was more evident when treating the cells with 7-hydroxy-3,4-dihydrocadalene at IC_50_ concentration. Slightly differences of Bcl-2 levels were observed but not significant.

### Effect on mitochondrial bioenergetic metabolism

**Figure 6:**
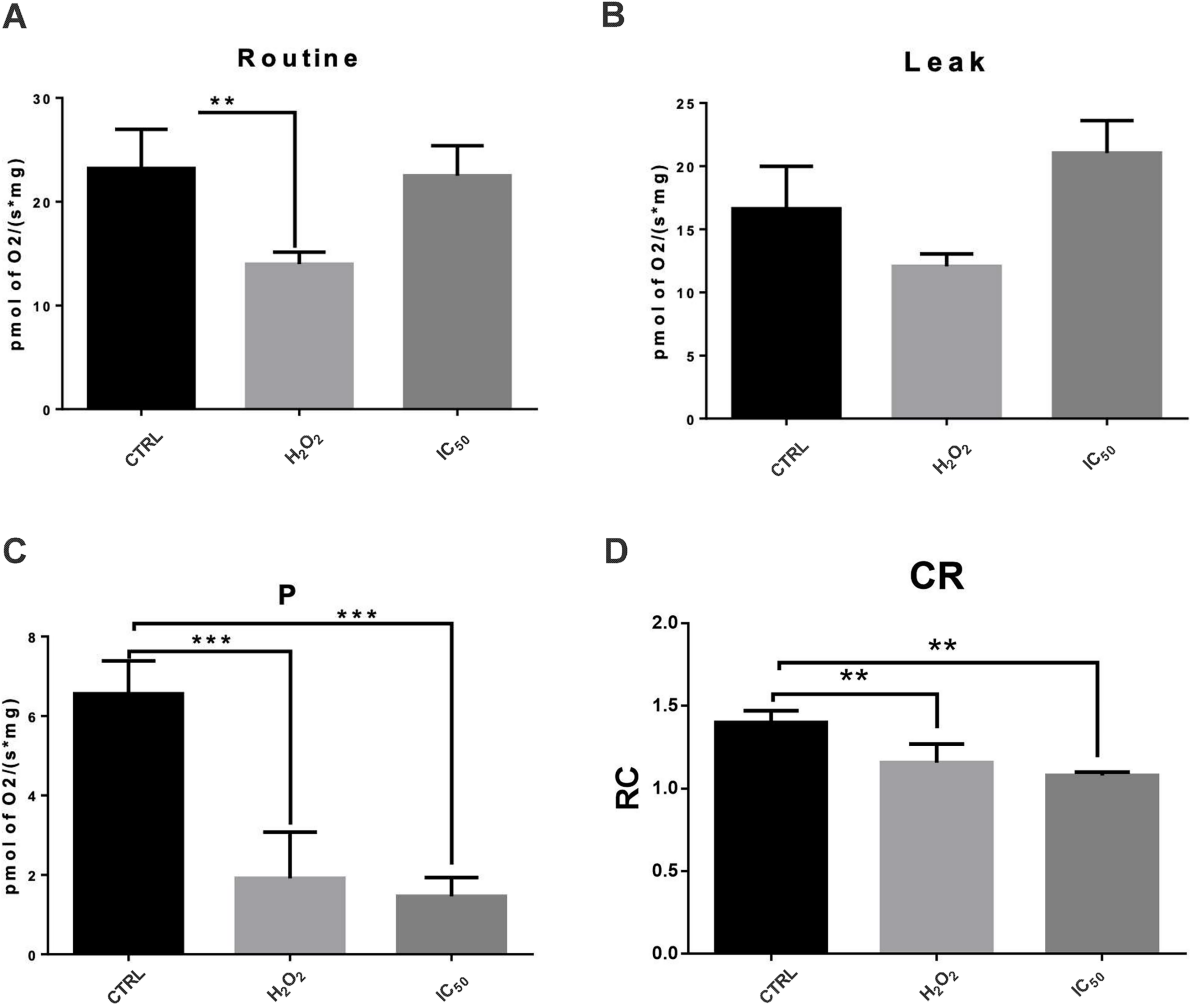
Effect of 7-hydroxy-3,4-dihydrocadalene on mitochondrial respiratory parameters: (A) routine, (B) leak, (C) OXPHOS associated respiration (P), and (D) control index (CI). MCF7 cells were treated with 7-hydroxy-3,4-dihydrocadalene (IC_50_) and hydrogen peroxide 1.0 mM as a positive control for 48 h. Data are expressed as the mean ± SD. Dunnet’s test **P < 0.01 and ***P < 0.001 *vs*. control, *n* = 5–7.

To determine if ROS production increases is related to mitochondrial bioenergetic metabolism alterations, the respiratory parameters including routine and leak respirations, respiration attributable to OXPHOS (P) and RCI were evaluated. As shown in Fig. 6, MCF7 cells were treated with 7-hydroxy-3,4-dihydrocadalene at IC_50_ for 48 h and a drastically decreased in P and slightly reduction in RCI were observed. This reduction was more marked than that produced by H_2_O_2_ treatment.

## Discussion

MCF7 cells are the most frequent used *in vitro* models of human breast adenocarcinoma; they are estrogen-receptor positive and progesterone-receptor-positive, and they express characteristics of breast epithelial cells (Comşa, Cîmpean & Raica, 2015).

Reactive oxygen species (ROS) play a dual role in cancer. On one hand, they are involved in initiation, promotion, and progression of cancer. On the other hand, ROS have anticancer activities by inducing apoptosis and senescence, cell cycle arrest and inhibiting angiogenesis (Stojnev, Ristić-Petrović & Janković-Velicković, 2013; Glasauer & Chandel, 2014; Galadari et al., 2017). Certain chemotherapeutic agents commercially available such as paclitaxel (Alexandre et al., 2007) and 5-fluorouracil (Afzal et al., 2012) induce the generation of ROS in cancer cells. Moreover, there have been identified several anticancer ROS-production compounds from natural origin including cribrostatin 6, D,L-sulforaphane and tanshinone IIA, among others (Barrera, 2012). Therefore, the use of prooxidants agents that increase the levels of ROS are a promising therapeutic target in the treatment of cancer. Then, we investigated the potential cytotoxic properties *via* oxidative stress of the natural product 7-hydroxy-3,4-dihydrocadalene and the semi-synthetic cadinanes derivatives 7-(phenylcarbamate)-3,4-dihydrocadalene and 7-(phenylcarbamate)-cadalene against breast cancer cells using (Z)-4-hydroxytamoxifen as a positive cytotoxic control since is a Selective Estrogen Receptor modulator (SERM) that has remained the antihormonal therapy of choice for the treatment of Estrogen Receptor (ER) positive breast cancer. The molecular subtype of MCF7 cell line is Luminal A which exhibits high expression of ER. Hassan et al. (2018) reported an IC_50_ = 4.51 μg/mL (12.12 μM) in MCF7 cell line, meanwhile the research group of this work found a IC_50_ = 16.50 μM in the same cell line.

The natural product showed higher cytotoxic potency (IC_50_ = 55.24 μM) than the semisynthetic derivatives 7-(phenylcarbamate)-3,4-dihydrocadalene and 7-(phenylcarbamate)-cadalene which showed lower or null cytotoxic potential. Previously, according to Egas et al. (2017), both semisynthetic compounds are highly cytotoxic on some human cell lines including MCF7. However, those compounds were dissolved in DMSO and cytotoxic assay was sulforhodamine B (SRB), meanwhile in our experimental conditions, the compounds were dissolved in phosphate-buffered saline (PBS) and the cytotoxic assay was thiazolyl blue tetrazolium bromide (MTT).

Poor pharmacokinetics is one of the main limitations or therapeutic failures in the research and development of new drugs. Absorption, distribution, metabolism, and elimination (ADME) related key physicochemical properties have been described for anticancer agents to have optimal bioavailability and non-toxicity (Lipinski et al., 2001; Ghose, Viswanadhan & Wendoloski, 1999; Veber et al., 2002; Singh, 2016). Therefore, using *in silico* studies, we predicted different physicochemical characteristics that limited anticancer activity including molecular weight, lipophilicity (log P), aqueous solubility (log S), hydrogen bond acceptor (HBA), hydrogen bond donor (HBD), topological polar surface area (TPSA), number of rotatable bonds (nRot) and distribution coefficient at pH = 7.4 (log D). Singh (2016) established that the optimal physico-chemical of anticancer compounds to have ideal ADME properties were 200 < *M*_*W*_ ≤ 800, 1 < LogP ≤ 5, −6 ≤ LogS ≤ −1, 5 ≤ HBA ≤ 13, 1 ≤ HBD ≤ 5, 50 ≤ TPSA ≤ 180, 0 ≤ n_Rot_ ≤ 10 and logD = 2.8. Moreover, Lipinski et al. (2001), considered *M*_*W*_ < 500, LogP < 5, HBA < 10 and HBD < 5. Furthermore, for Veber et al. (2002) was essential TPSA ≤ 140 and n_Rot_ ≤ 10 and Ghose, Viswanadhan & Wendoloski (1999) attributed certain importance to molecular weight and lipophilicity (160 ≤ *M*_*W*_ ≤ 480 and 0.4 ≤ LogP ≤ 5.6). Regarding the assayed compounds, the semisynthetic cadalenes derivatives do not have three of the cited rules for two physical-chemical parameters (logP and logS) neither one of the rules related to the number of hydrogen acceptors (HBA). Therefore, this *in silico* study suggests that the natural product 7-hydroxy-3,4-dihydrocadalene has better physico-chemical properties to be a promising cytotoxic agent than the two semi-synthetic compounds 7-(phenylcarbamate)-3,4-dihydrocadalene and 7-(phenylcarbamate)-cadalene. These semisynthetic cadalenes derivatives are structurally characterized as having a phenylcarbamate group in the carbon 7. Currently there are several antitumor agents with carbamate groups such as irinotecan, mitomycin C, entinostat and capecitabine with good pharmacokinetics properties, ability to pass through the cell membranes and modulate intra and intermolecular interactions with effector proteins and receptors (Ghosh & Brindisi, 2015). However, in our study we demonstrated that the phenolic OH of the natural product 7-hydroxy-3,4-dihydrocadalene seems to be essential for the cytotoxic activity of these compounds. These findings support similar results obtained by Lee et al. (2007).

Since the semisynthetic derivatives 7-(phenylcarbamate)-3,4-dihydrocadalene and 7-(phenylcarbamate)-cadalene were found not cytotoxic on MCF7 cells at the established experimental conditions and the *in silico* study shown that these both compounds do not present the best physical-chemical conditions to be good cytotoxic candidates, we decided to continue the study only with the natural compound 7-hydroxy-3,4-dihydrocadalene. Previously, a screening of potential cytotoxic compounds derived from *H. inuloides* (including 7-hydroxy-3,4-dihydrocadalene) was performed against three cell lines (K562, HCT-15 & MFC7) by Rodríguez-Chávez et al. (2015b). They found a 7-hydroxy-3,4-dihydrocadalene IC_50_ of 61.37 µM against MCF7 cell line. However, a potential mechanism of action which7-hydroxy-3,4-dihydrocadalene exerted cytotoxicity against MCF7 was not investigated.

The natural product 7-hydroxy-3,4-dihydrocadalene at IC_50_ concentration significantly increased the intracellular ROS levels. Hydrogen peroxide was used as positive control. H_2_O_2_ is a non-radical specie that easily diffuses across cell membranes and produces hydroxyl radical which is one of the most harmful reactive species known. *In vivo* studies have demonstrated that H_2_O_2_ decreases tumor growth (Nathan & Cohn, 1981; Uetaki et al., 2015).

ROS attack carbon-carbon double bond of polyunsaturated fatty acids in cell membranes, leading to changes in its permeability, fluidity and consequently modifying cell integrity (Barrera, 2012). Cell membranes are common target for the free radicals like hydroxyl radical (HO^•^) and hydroperoxyl (HO^•^_2_), generating MDA as one of the main products of lipid peroxidation. The TBARS test is widely used to measure MDA concentration (Esterbauer & Cheeseman, 1990). Our results suggest that lipid peroxidation production can induce one of the principal ways of the cytotoxic action of the natural product 7-hydroxy-3,4-dihydrocadalene. It is interesting to note the higher lipid peroxidation produced after 7-hydroxy-3,4-dihydrocadalene treatment than H_2_O_2_ treatment, even when H_2_O_2_ produced higher levels of ROS in DCFH-DA assay. Therefore, it can be suggested that different and more aggressive reactive species than hydroxyl radical were produced by 7-hydroxy-3,4-dihydrocadalene. However, the techniques used in this work are not capable of identifying any species or free radicals. DCFH-DA assay was used to quantify intracellular ROS production and further research and techniques need to be implemented to differentiate them.

ROS are involved in cell apoptosis induction. Apoptotic cell death is a stress response to chemotherapeutic agents (Liu, Cheung & Che, 2006). Apoptosis is associated with the activation of caspase pathway. The intrinsic pathway implicates the release of cytochrome c from mitochondria and the activation of the initiator caspase 9, and consequently the executioner caspase 3 (Pfeffer & Singh, 2018). Moreover, the stimulation of proapoptotic molecules such as caspase 3 and the inhibition of antiapoptotic molecules such as Bcl-2 are common therapeutically strategies to induce cancer cell death (Pfeffer & Singh, 2018). The observed increase in caspases-3/9 activity and caspase 3 level (by immunoblotting) suggest an apoptotic event since those are some of the main final downstream effectors/triggers to programmed cell death process. It is proposed that the cytotoxic activity of 7-hydroxy-3,4-dihydrocadalene is mainly due to the pro-oxidant action and that this may be the mechanism of action involved in cell death by apoptosis given the cross-linking of ROS with different signaling pathways (Kaminskyy & Zhivotovsky, 2014; Redza-Dutordoir & Averill-Bates, 2016; Galadari et al., 2017). Additionally, the morphological features observed suggest an apoptotic process. Some of the morphological changes seen, such as rounding-up of the cell and plasma membrane blebbing, after 7-hydroxy-3,4-dihydrocadalene and H_2_O_2_ treatments, are well stipulated criteria to define apoptosis (Kerr, Wyllie & Currie, 1972; Kroemer et al., 2009).

Mitochondria is essential to induce and maintain the tumoral profile in many cancer cells by performing bioenergetic and biosynthetic processes. Furthermore, it is considered as one of the main ROS production sites in cells and it can participate in apoptotic cell death regulation (Zong, Rabinowitz & White, 2016). It has been reported that mitochondrial decoupling promotes ROS production, which contributes to oxidative stress and to cell damage (Zorov, Juhaszova & Sollott, 2014). Our results indicate that 7-hydroxy-3,4-dihydrocadalene significantly reduced mitochondrial ATP synthesis and induced mitochondrial uncoupling. Both dysfunctions, oxidative stress and energetic disturbance may be triggering apoptotic pathway in these cells.

## Conclusions

In conclusion, our results suggest that the incorporation of the phenylcarbamate group at carbon number seven of the natural compounds 7-hydroxy-3,4-dihydrocadalene and 7-hydroxycadalene, isolated from *Heterotheca inuloides*, do not increase their cytotoxic effect on the cell line MCF7. The phenolic OH of 7-hydroxy-3,4-dihydrocadalene seems to be essential for the time- and concentration-dependent cytotoxic activity against MCF7 cell line in addition to being capable of decreasing proliferation and migration. Additionally, the induction of oxidative stress is proposed as the potential mechanism of action responsible for promoting cell death apparently apoptosis-mediated by a significant increase of activity and levels of final effectors implicated on cell programmed cell death.

Considering that 7-hydroxy-3,4-dihydrocadalene is able of promoting the generation of reactive oxygen species in the MCF7 cell line and a consequent cytotoxic effect, it would be interesting to evaluate whether this natural compound can exert its cytotoxic effect against other different malignant cell lines.

Future research should aim to design synthetic analogues with phenolic OH of 7-hydroxy-3,4-dihydrocadalene to improve the cytotoxic potency and to evaluate its activity in *in vivo* models to be used as a pro-oxidant adjuvant or coadjuvant for antitumoral therapies.

## Supplemental Information

10.7717/peerj.15586/supp-1 Supplemental Information 1 Raw Data.

10.7717/peerj.15586/supp-2 Supplemental Information 2 Effect on cell viability. MCF7 human breast cancer cells were treated with different concentrations from 0.1 to 100 µM for 48, and 72 h with: (A) 7-hydroxy-3,4-dihydrocadalene. Two main IC_50_ were found: 55.24 & 52.83 μM (for 48 & 72 h, respectively). Since no significant difference was found between these IC_50_, we decided to employ IC_50_ of 48 h to reduce time in subsequent experiments.

10.7717/peerj.15586/supp-3 Supplemental Information 3 WB gels.

## Additional Information and Declarations

### Data Availability

The following information was supplied regarding data availability: The raw data are available in the Supplemental Files.
