# Supplementary figures and images for: The cytotoxicity effect of 7-hydroxy-3,4-dihydrocadalene from Heterotheca inuloides and semisynthetic cadalenes derivates towards breast cancer cells: involvement of oxidative stress-mediated apoptosis
Source: PeerJ. 2023 Jun 20;11:e15586. doi: 10.7717/peerj.15586 (PMC10289085; doi:10.7717/peerj.15586)

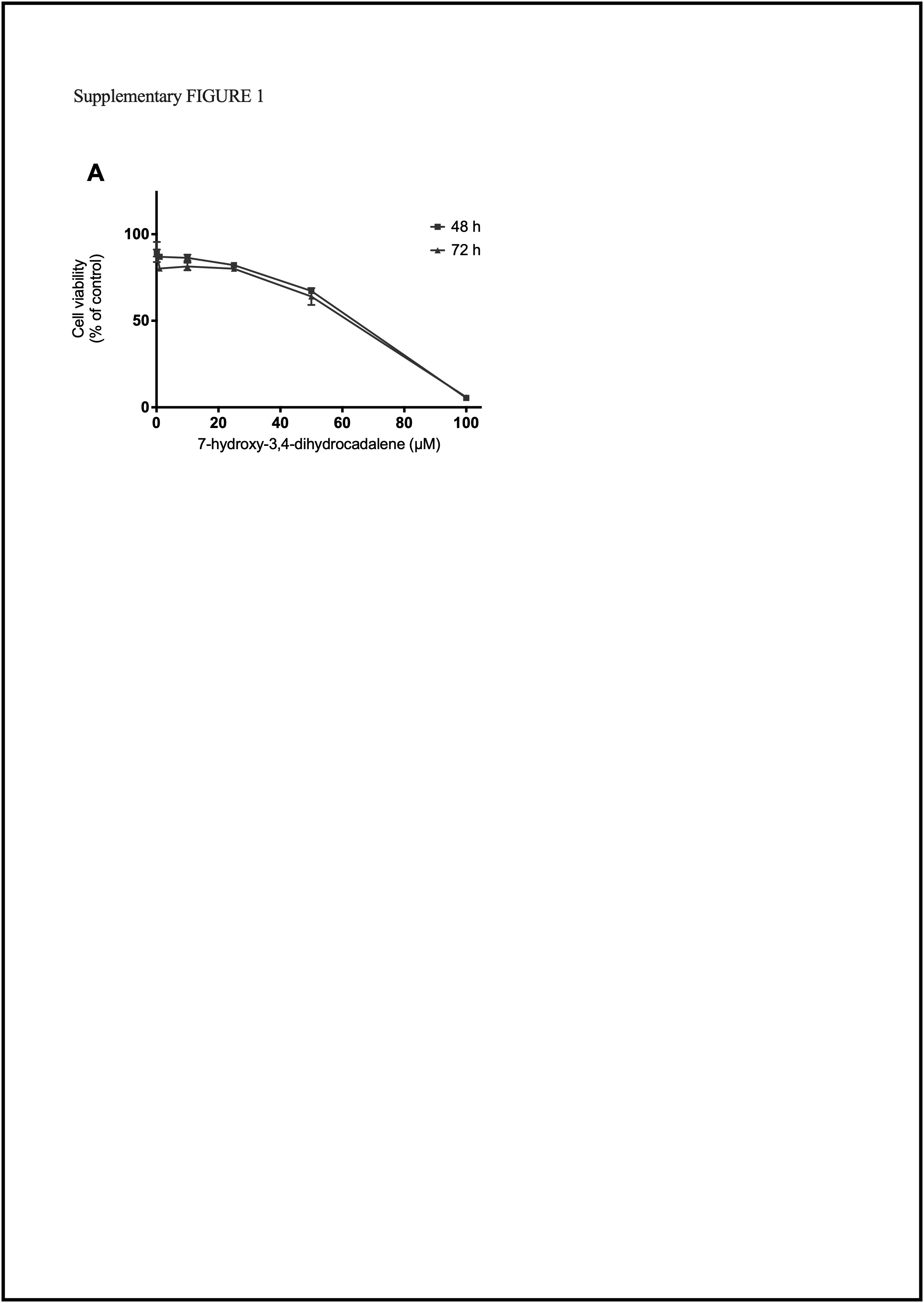

Supplement: Supplemental Information 2 — MCF7 human breast cancer cells were treated with different concentrations from 0.1 to 100 µM for 48, and 72 h with: (A) 7-hydroxy-3,4-dihydrocadalene. Two main IC50 were found: 55.24 & 52.83 μM (for 48 & 72 h, respectively). Since no significant difference was found between these IC50, we decided to employ IC50 of 48 h to reduce time in subsequent experiments. [file peerj-11-15586-s002.jpg]
