# Supplementary material for: The cytotoxicity effect of 7-hydroxy-3,4-dihydrocadalene from Heterotheca inuloides and semisynthetic cadalenes derivates towards breast cancer cells: involvement of oxidative stress-mediated apoptosis
Source: PeerJ. 2023 Jun 20;11:e15586. doi: 10.7717/peerj.15586 (PMC10289085; doi:10.7717/peerj.15586)
Supplement: Supplemental Information 3 [file peerj-11-15586-s003.zip › WBNew.pptx]

## Slide 1
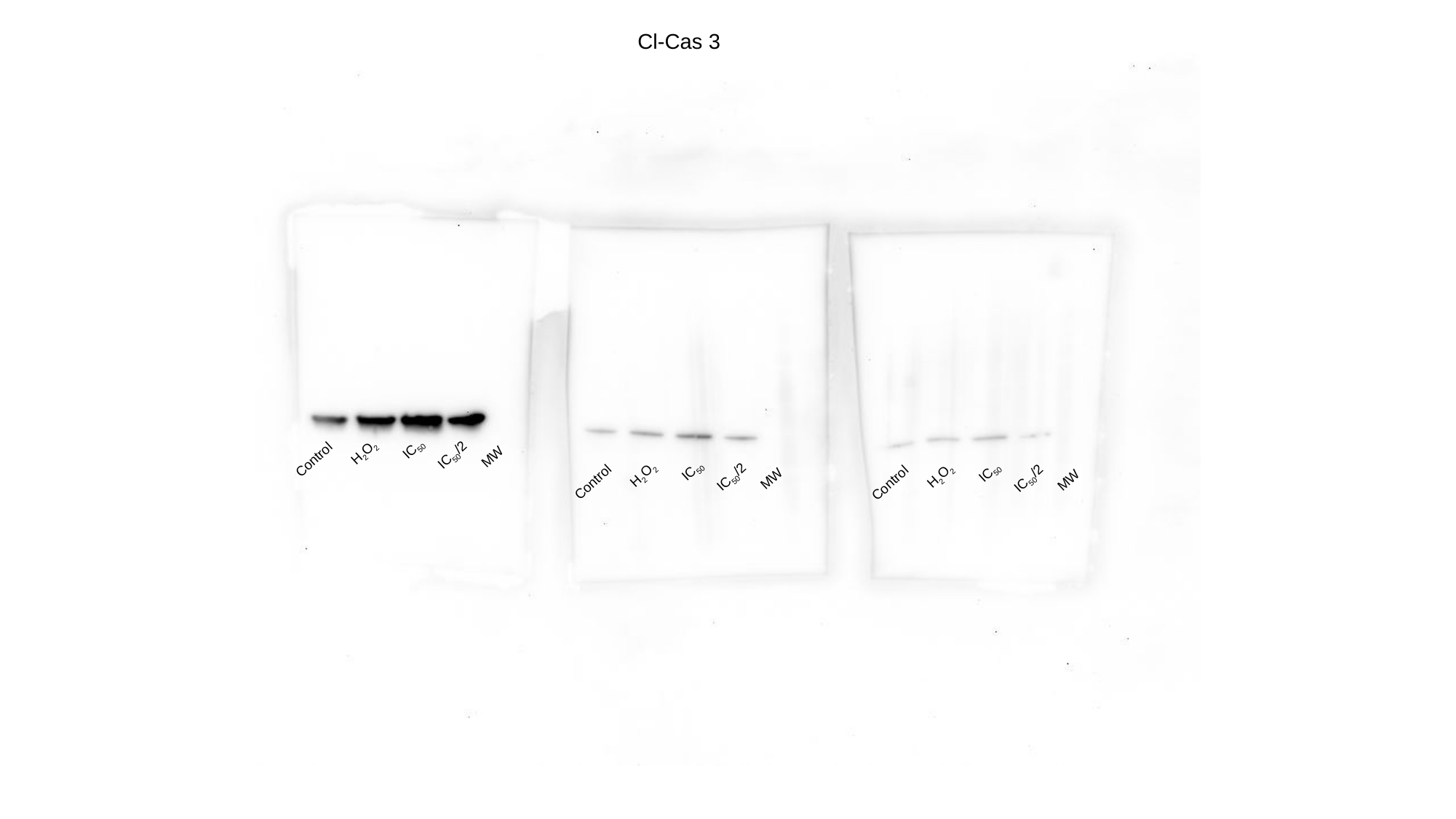

Cl-Cas 3
IC50
H2O2
IC50/2
MW
Control
IC50
IC50
H2O2
H2O2
IC50/2
IC50/2
MW
MW
Control
Control

## Slide 2
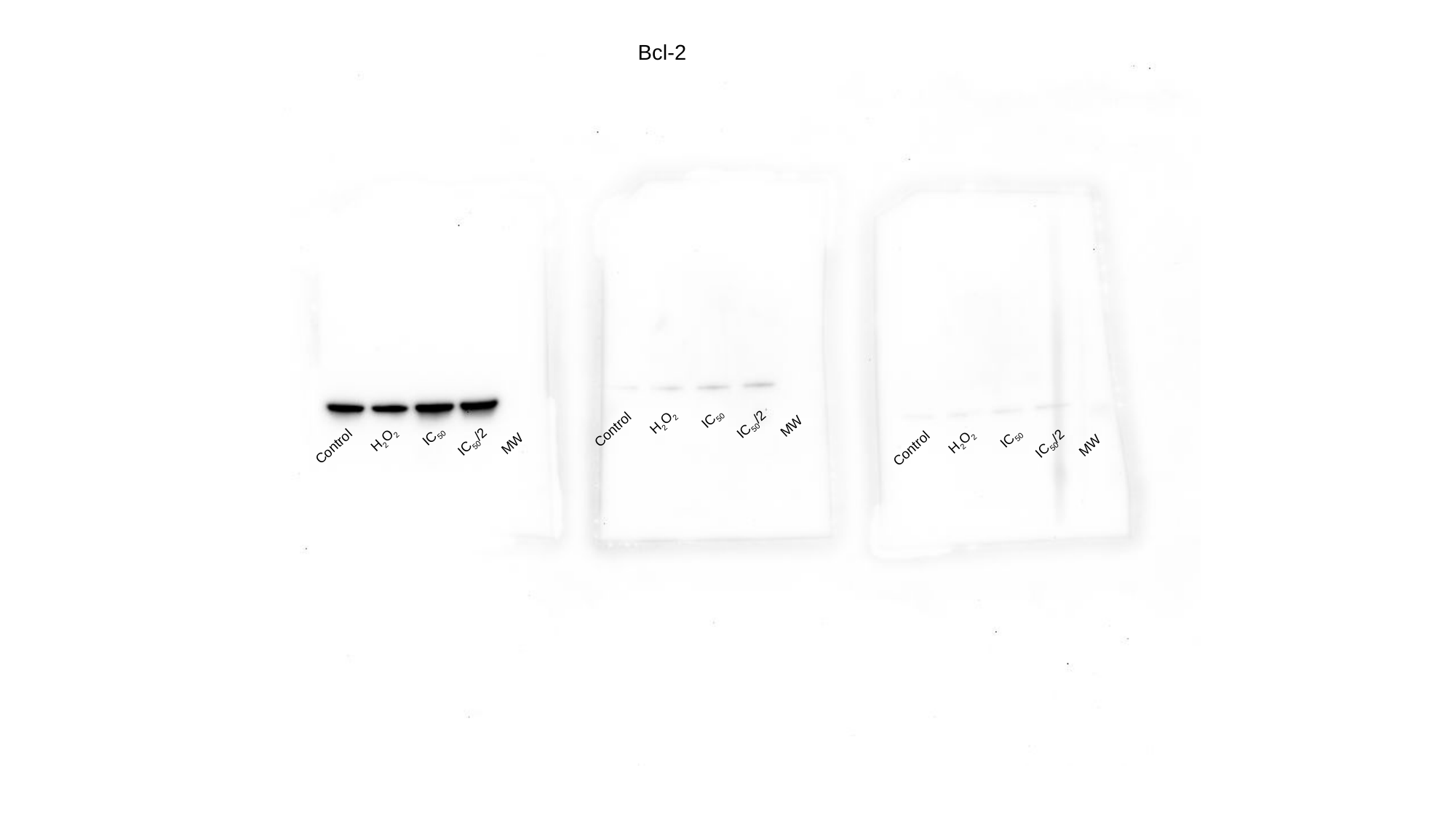

Bcl-2
IC50
H2O2
IC50/2
MW
Control
IC50
IC50
H2O2
H2O2
IC50/2
IC50/2
MW
MW
Control
Control

## Slide 3
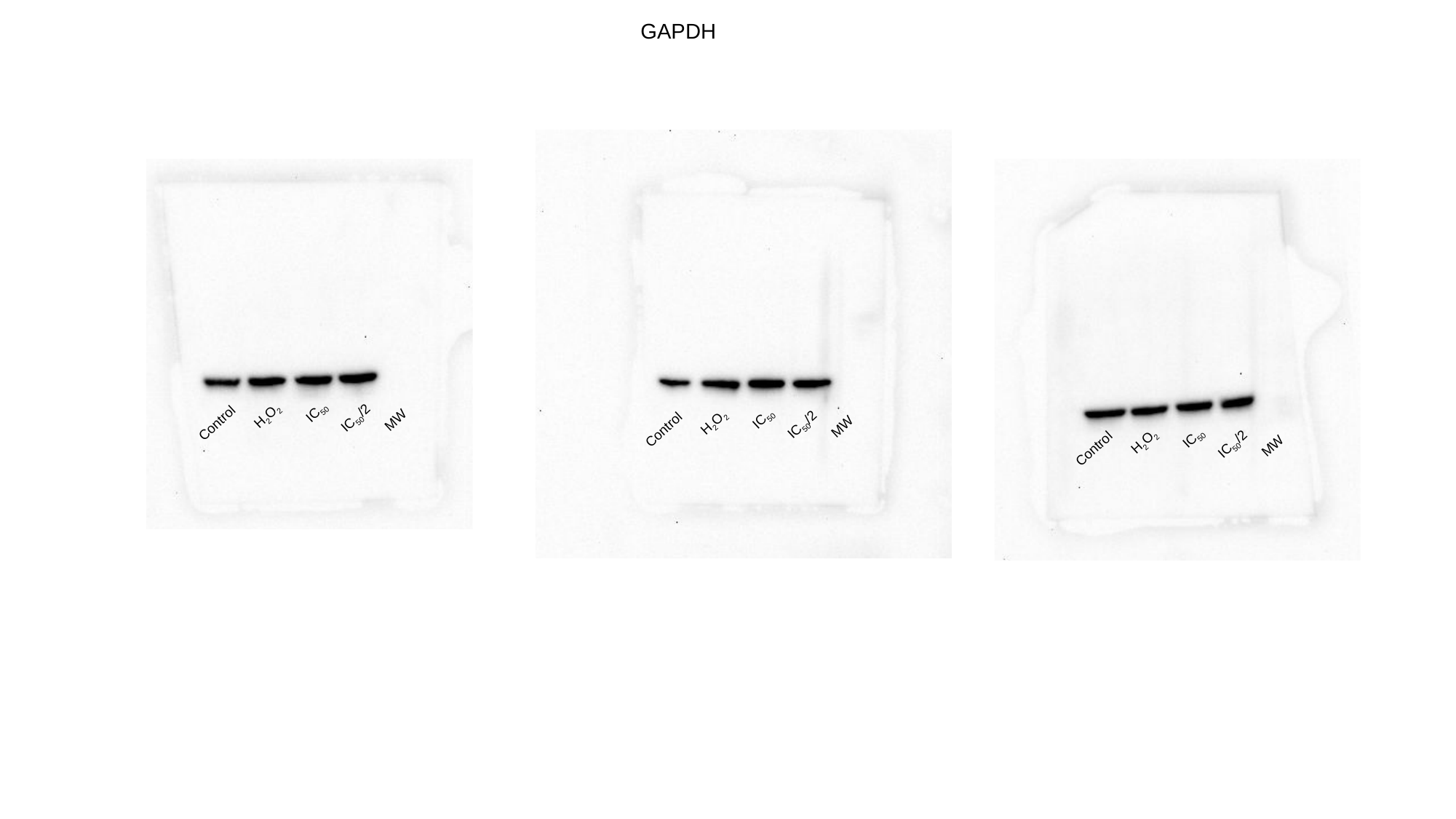

GAPDH
IC50
H2O2
IC50/2
IC50
MW
H2O2
Control
IC50/2
MW
Control
IC50
H2O2
IC50/2
MW
Control
